# Acute myelitis with multicranial neuritis caused by Varicella zoster virus: a case report

**DOI:** 10.1186/s12883-022-02571-y

**Published:** 2022-02-05

**Authors:** Qi Liu, Xiaomeng Zhou, Zhenfei Li

**Affiliations:** 1 Department of Neurology, The Second Hospital of Hebei Medical University, Shijiazhuang, 050000 PR China; 2 Key Laboratory of Neurology of Hebei Province, The Second Hospital of Hebei Medical University, 215 Heping West Road, Shijiazhuang, 050000 PR China

**Keywords:** Varicella zoster virus, Central nervous system, Acute transverse myelitis, Multicranial neuritis, Metagenomic next-generation sequencing

## Abstract

**Background:**

Varicella zoster virus (VZV) can remain lifelong in the latent state in ganglionic neurons and adrenal glands after the initial infection. However, it can be reactivated anytime and can trigger several severe neurological manifestations such as encephalitis, meningitis, Ramsay-Hunt syndrome, cerebellitis, myelitis, and stroke. In addition, due to the diversity of clinical manifestations, clinical diagnosis of VZV can be difficult, especially in the absence of varicella. Here, we describe the case of a 52-year-old male who presented with symptoms of acute myelitis as well as polycranial neuritis, and was finally diagnosed with VZV infection through metagenomic next-generation sequencing (mNGS).

**Case presentation:**

A 52-year-old male came to our hospital with complaint of headache, fever, weakness of right lower limb, abdominal distension, and hearing loss. T2-weighted MRI revealed a hyperintense lesion in the spinal cord extending from T8 to T11. In addition, enhanced MRI showed small patches and strips hyperintensities in both the spinal cord and meninges. Plain abdominal radiographs and abdominal computed tomography (CT) scan displayed air-fluid levels and incomplete bowel obstruction. Moreover, electrophysiological evaluation of the peripheral neuropathy in the extremities was found to be normal. Finally, by using metagenomic next-generation sequencing (mNGS) we found that the copy number of VZV DNA in cerebrospinal fluid (CSF) was significantly increased and IgG antibody against VZV in CSF was also noted to be positive. Hence, VZV infection was identified in patient’s central neuron system. Finally, after a few days of low dose steroid treatment, the patient's symptoms were found to be significantly improved.

**Conclusions:**

The findings indicate that we should pay proper attention to the various symptoms caused by VZV infection due to the clinical heterogeneity, especially in the absence of cutaneous lesions.

## Background

Varicella Zoster Virus (VZV) is a neurotropic DNA virus. Varicella (chickenpox) is the usual initial symptom, and then VZV can exist in a latent state in the ganglionic neurons and adrenal glands [1]. In adult, VZV reactivation is often accompanied by a significant decline of cell-mediated immunity, which can result in attack on the both peripheral and central nervous systems. This can lead to a variety of neurological diseases including meningitis, encephalitis, cerebral vasculopathies, cranial nerve inflammation, Guillain-Barré syndrome (GBS), other peripheral nervous system (PNS) diseases, and postherpetic neuralgia (PHN) [1, 2] It has been found that for the non-specific and indistinguishable neurological manifestation from other viral infections, it is difficult to relate the neurological syndromes to VZV in the absence of distinct cutaneous lesions [3].

A few previous studies have been conducted on the global epidemiology of the neurological manifestations in VZV but the findings remain unclear. The various current studies are mainly regional studies or case reports. PHN is considered to be the most common complication of VZV, which can occur in ~ 15% of cases, and diagnosed in 40% of the patients over 60 years of age [3, 4]. VZV is still recognized as the most or second common infectious etiology of meningitis and encephalitis in adults, contributing 4.4–24% and 12–27.5% cases, respectively [5–7]. VZV infection can account for 8–28% of Bell’s palsy (BP) cases and 15.2% of cranial neuritis cases. VZV infection may trigger GBS, accounting for 11.1% of cases [7, 8]. However, exact epidemiological data related to VZV vasculopathy and myelopathy are still unknown, and generally they are considered to be less than 10% [7]. VZV myelopathy can present itself as a self-limiting or sometimes fatal myelitis, and mostly occurs in immunocompromised individuals [3]. Our case highlights a previously healthy patient with VZV myelitis and polycranial neuritis without displaying any rash.

## Case presentation

A 52-year-old man was admitted to our hospital for headache, fever, weakness of the right lower limb, abdominal distension, and hearing loss. He was generally in good health without any medical history before. Initially, the patient experienced headache and fever, with a maximum temperature of 39 °C. Two days later, he also developed bilateral facial paralysis, right leg weakness, and bilateral hearing loss. Thereafter, two more days later, new symptoms including that of abdominal distention and urinary retention occurred. He did not experience any rash.

On physical examination, he was found to be alert and oriented. The right pupil was dilated (3 mm VS. 2.5 mm) but the direct and indirect light reflection was slow, without ocular movement disorder, thereby indicating incomplete injuries of the right oculomotor nerve. Bilateral peripheral facial paralysis and presence of hearing loss suggested that his bilateral facial nerves and vestibulocochlear nerves were affected. In addition, the strength of his right lower limb appeared to be significantly weak (medical research council (MRC) grade 3/5), while the left lower limb and both upper limbs were found to be normal ( (MRC) grade 5/5). It was observed that though bilateral ankle and knee jerk reflexes disappeared, whereas Babinski’s sign was positive on the right side. Furthermore, the puncture sensation below the T6 level on the left side of the body decreased markedly, while the sensation of vibration and touch were found to be normal. In addition, combined with abdominal distention and urinary retention, it was suspected that the patient suffered from a condition with spinal cord involvement.

**Fig. 1:**
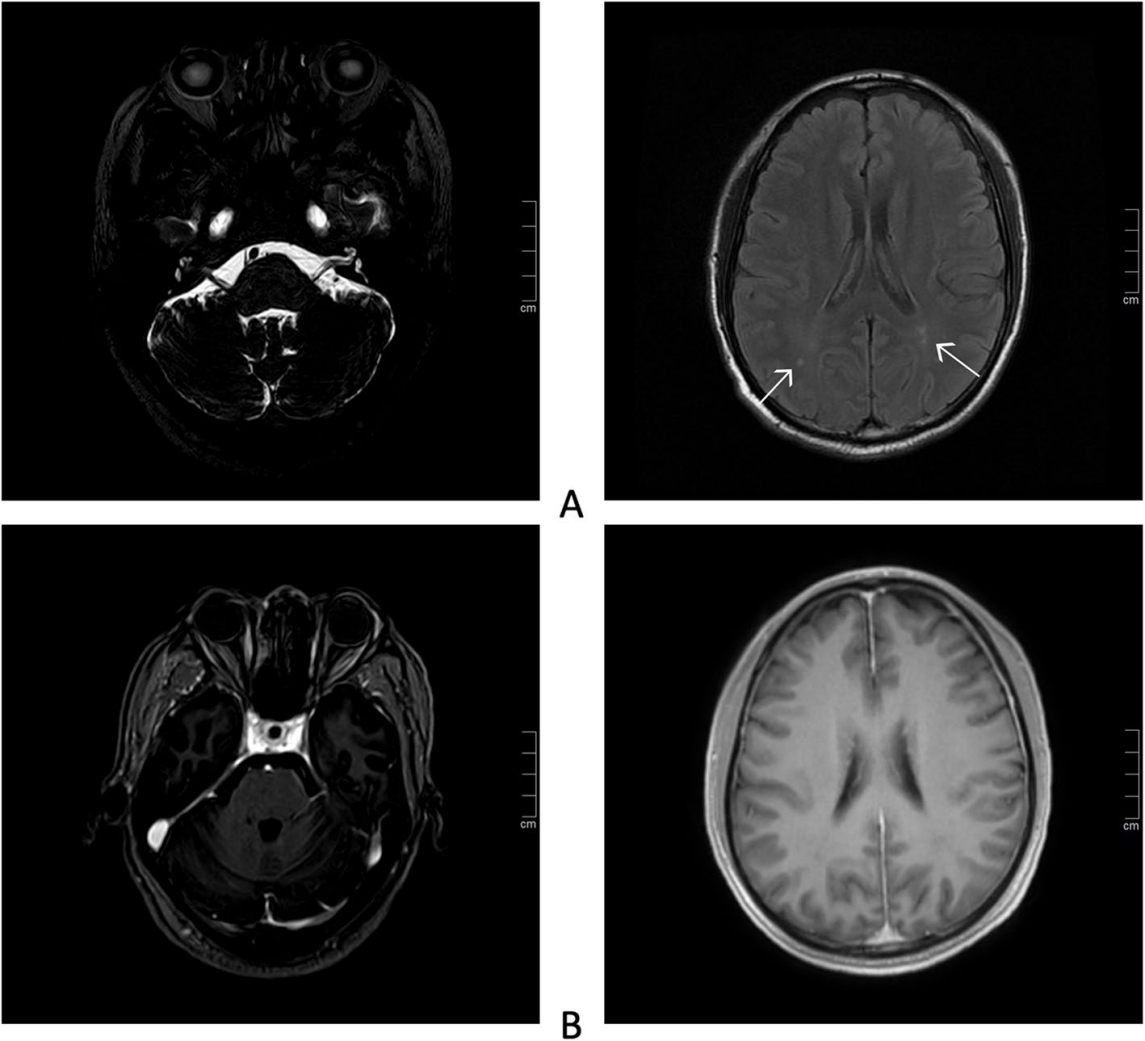
Brain MRI showed non-specific white matter changes but cranial nerve MRI was normal, with no observed in enhancement signal in contrast-enhanced scans. **A** Plain MRI of brain and cranial nerve. White arrows indicate non-specific white matter changes; **B** Contrast-enhanced MRI scan of brain and cranial nerve

**Fig. 2:**
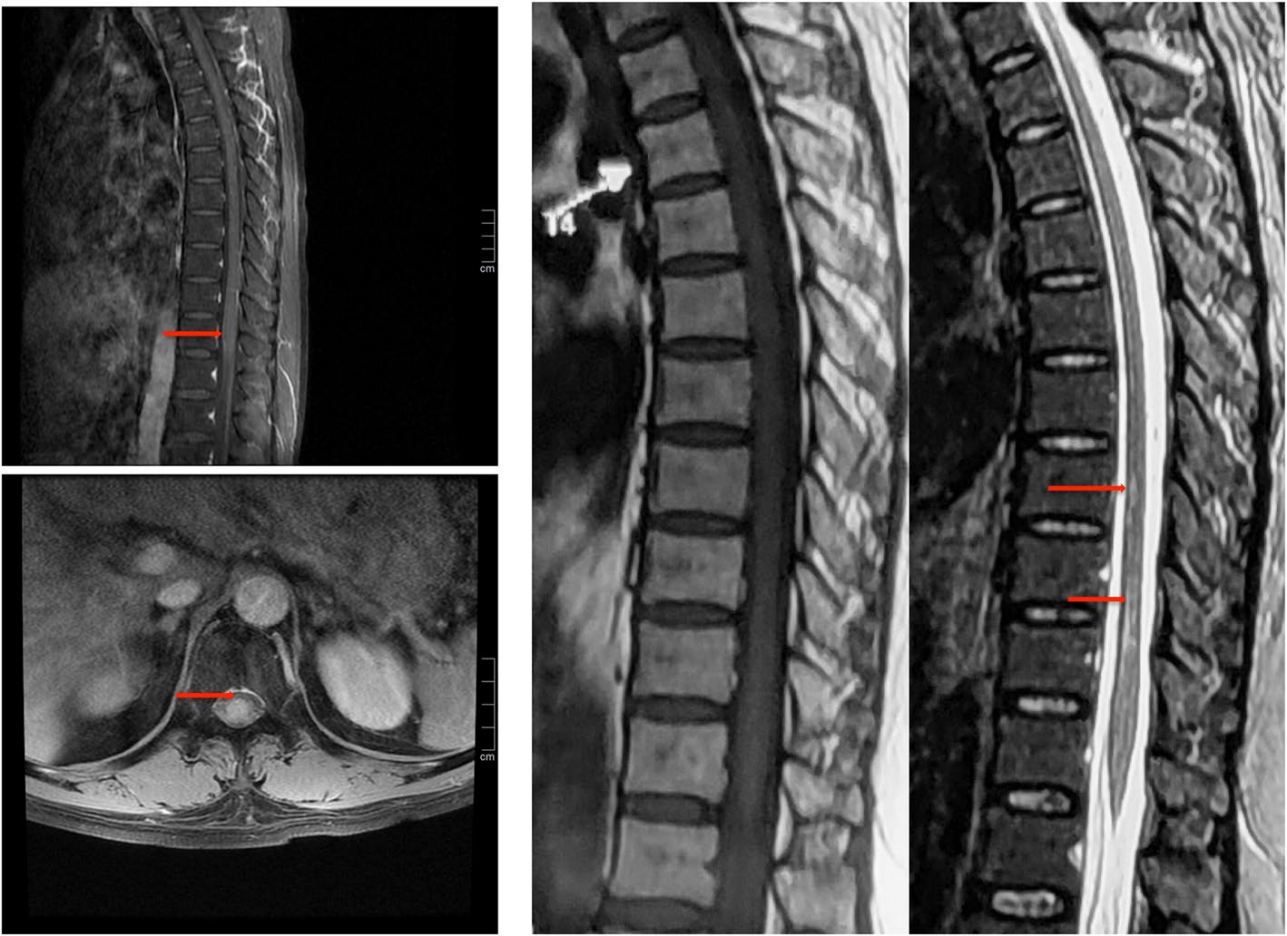
Spinal MRI revealed a hyperintense T2 lesion in the spinal cord extending from T8 to T11, and enhancement MRI indicated small patchy and strips hyperintensities in the spinal cord and meninges. The red arrow refer to the injuries

**Fig. 3:**
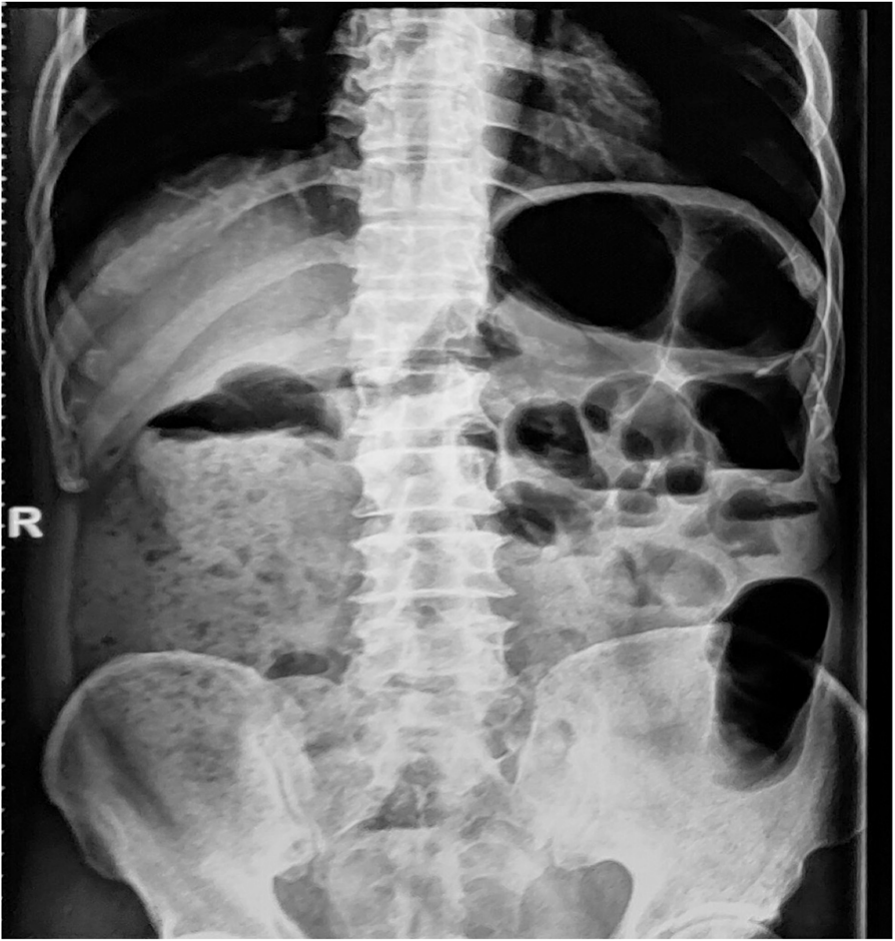
Plain X-rays and abdominal computed tomography scan demonstrated air-fluid levels and an incomplete obstruction of the colon

**Fig. 4:**
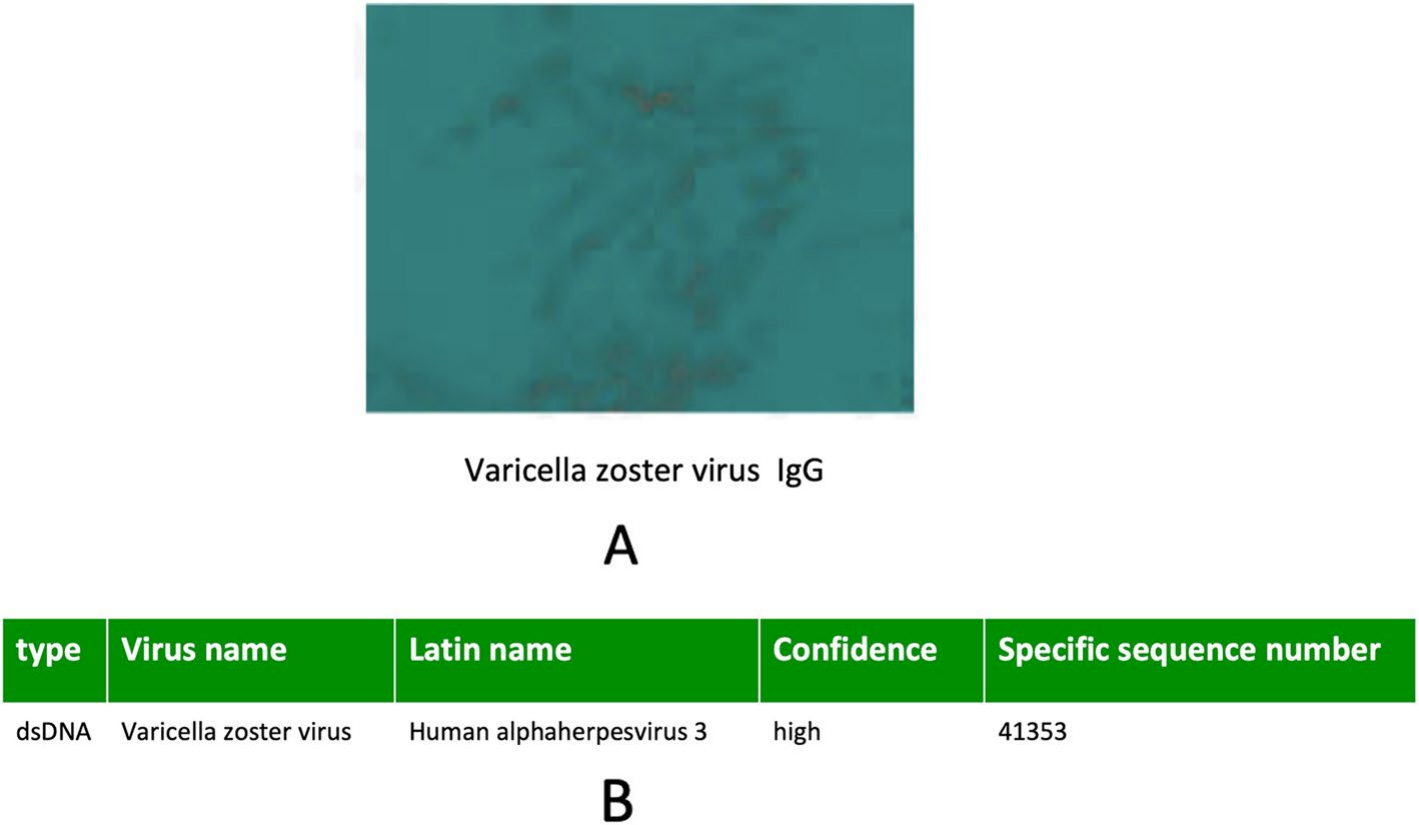
Metagenomics next-generation sequencing (mNGS) of CSF displayed a significant increase of VZV DNA, and VZV-specific intrathecal antibodies test was also found to be positive

The physical examination revealed that the patient had multiple cranial nerves and spinal cord injuries. Hence, neurological infection was primarily suspected. Normal nerve conduction studies ruled out the occurrence of PNS. Routine CSF analysis displayed an increased cell count to 272/mm^3^ (normal < 5/mm ^3^), of which lymphocytes accounted for 99%, and a slightly elevated protein levels to 199 mg/dl (normal < 45 mg/dl). Moreover, CSF cytology revealed primarily a lymphocyte reaction. Lyme titer, India ink capsule staining and interferon-gamma Release Assay (IGRA) were all found to be negative. Brain MRI showed mild non-specific white matter changes and cranial nerve MRI was found to be normal, with no enhancement signal in the contrast-enhanced scans (Fig. 1). In addition, cell count was not found to be significantly enhanced in CSF analysis, which ruled out acute disseminated encephalomyelitis (ADEM). T2-weighted MRI revealed a hyperintense lesion in the spinal cord extending from T8 to T11 (Fig. 2), enhanced MRI showed small patchy and strips hyperintensities in spinal cord and meninges (Fig. 2). Moreover, plain abdominal radiographs and abdominal computed tomography (CT) scan displayed air-fluid levels and incomplete bowel obstruction (Fig. 3). In addition, as limited evidence was obtained from the routine laboratory investigations including immunological examinations and CSF analysis, we next performed metagenomic next-generation sequencing (mNGS) to distinguish *Listeria monocytogenes* and genotyping of the virus. The result of mNGS in CSF showed a significant increase of VZV DNAc(41,353 reads) (Fig. 4). Furthermore, we performed a VZV-specific intrathecal antibodies test and the result was also noted to be positive. Ultimately, we diagnosed that both polyneuritis cranialis and myelitis were caused by VZV infection. The diagnosis of intestinal pseudo-obstruction (paralytic ileus) was secondary to the segmental paresis caused by VZV infection. After intravenous administration of ganciclovir 5 mg/kg q12hr, combined with 5 mg/d dexamethasone therapy, and conservative managements such as nasogastric tube decompression, fasting, as well as intravenous (IV) resuscitation, the patient’s abdominal distention was noted to be gradually improved and disappeared within 14 days. The issues of bilateral facial paralysis and hearing loss were also found to recover gradually within a month too. The strength of the right lower limb rehabilitated to grade of 4/5 (MRC). However, the urinary retention was not observed to improve significantly.

## Discussion and conclusions

Reactivation of VZV latent on the ganglia can cause different disorders, such as herpes zoster, meningitis or meningoencephalitis, cerebellum encephalitis, monocranial nerve palsy, polycranial nerves palsy, vascular disease, myelopathy and various other important ocular inflammatory diseases, which have been all reported before [2]. It should also be noted that sometimes VZV reactivation can also manifest multiple neurological disorders without rash [9], which poses a challenge for clinicians to identify it quickly and accurately.

VZV contributes to 15.2% of cranial neuritis cases, and cranial nerves VII, VIII have been found to be the most commonly involved. Polyneuritis cranialis (VII, VIII, IX, X) caused by VZV have also been described before [10, 11]. Moreover, previously, it was reported that a 41-year-old female with polyneuritis cranialis (III, VII, VIII) was diagnosed with VZV infection due to the positive detection of anti-VZV IgG in CSF [12]. In our case, the patient suffered from the multiple cranial nerve injuries, including that of the right oculomotor nerve, bilateral facial nerves and vestibulocochlear nerves (III, VII, VIII). However, the performance of cranial nerves injuries was found to be incomplete. For example, the III cranial nerve involvement only manifested itself as pupil dilation without causing abnormal eye movement, and the bilateral VIII cranial nerve injuries only affected hearing but no vestibular nerve was found to be involved. The characteristics of selective polyneuritis cranialis caused by VZV has been described in several previous case reports [10–12]. In addition, the peculiar feature that the brain MRI of VZV-induced polyneuritis cranialis is often negative has also been described before [12], which was consistent with our current findings. This feature helps to rule out the occurrence of both tuberculous meningitis and sarcoidosis.

In our case, the patient displayed intestinal as well as urine retention, and the physical examination found crossed spinothalamic loss on one side without the posterior column loss on the opposite side, and a hyperintense T2 lesion in the spinal cord extending from T8 to T11 in enhanced spinal MRI (Fig. 2). Thus, combined with the characteristics of CSF changes, the diagnosis of myelitis was established. VZV induced acute transverse myelitis (ATM) has been reported to rarely occur in the event of VZV reactivation [7, 13]. Myelitis has been reported to be caused by the direct viral invasion based on post-mortem findings about the diagnosis of Cowdry type A intranuclear inclusions in spinal root ganglia and positive immunostaining of VZV antigens [13–15]. In our case, the patient complained intestinal pseudo-obstruction which was rare in myelitis. VZV reactivation can cause intestinal pseudo-obstruction which can primarily result because of the involvement of dorsal root ganglion, spinal cord, and the small intestine bowel (Ogilvie’s syndrome) [16–20]. Since the ascending colon and descending colon are primarily innervated by the sympathetic fibers of T6-T12 spinal cord collateral, and it has been found that when the collaterals are involved in spinal cord lesions, they can effectively cause spastic and paralytic intestinal obstruction. For instance, William et. al. has reported a case of myelitis with spastic ileus as an initial manifestation [21]. Additionally, it has also been reported that pseudo intestinal obstruction caused by VZV might be due to visceral motor neuropathies [22, 23]. Unlike previous reports that only observed intestinal dysfunction, the patient in our case also displayed retention of urine simultaneously. Since the regulation of intestinal motility is primarily co-controlled by the external spinal autonomic nervous system and the independent internal intestinal nervous system, the recovery of intestinal obstruction has been observed to be significantly earlier than that of urinary retention.

In view of the severe symptoms such as multiple cranial neuritis and myelitis in the patient, and analysis of the result of CSF examination, which revealed an abnormal increase of protein and cell counts, we first considered the possibility of tuberculous meningitis or autoimmune disease, such as sarcoidosis. Steroids can significantly reduce the occurrence of immune inflammatory response and inflammation sequelae. To avoid the possibility of infection spread caused by pulse steroid therapy, a small dose of steroid (5 mg/d dexamethasone) was initially applied in this case. VZV has been found to be a causative factor for a broad spectrum of neurological diseases [3, 7]. However, most of the previously reported cases were observed to be either multiple cranial neuritis or myelitis. In our case, multiple cranial neuritis and myelitis occurred simultaneously, which was a rare event in the clinical practice. It may be related to the higher virus load. Finally, the correct diagnosis was made based on the metagenomics next-generation sequencing and VZV antibody detection in CSF. Antiviral therapy is the main treatment for controlling and treating VZV infection. In addition, corticosteroid treatment has also been applied in some cases with neurological complications. In view of the significant improvement in the clinical symptoms, pulse steroid therapy was no longer applied after the diagnosis of VZV infection in our case. Ultimately, the patients received intravenous ganciclovir combined with 5 mg/d dexamethasone. Although there was sequelae in the form of bladder incontinence by the time the patient was discharged from the hospital, it was observed to be gradually improved during our follow-up.

VZV infection may be fatal, and hence early as well as accurate diagnosis and treatment can reduce both the associated disability and mortality. We should pay proper attention to the various symptoms caused by VZV infection due to the clinical heterogeneity, especially in the absence of cutaneous lesions. Hence, comprehensive examinations should be carried out to lower misdiagnosis or missed diagnosis and provide suitable treatment to the patients.

## Data Availability

All data generated or analyzed during this study are available from the corresponding author on reasonable request.

## Abbreviations

VZV: Varicella zoster virus
mNGS: Metagenomic next-generation sequencing
CSF: Cerebrospinal fluid
PNS: Peripheral nervous system
PHN: Ostherpetic neuralgia
BP: Bell’s palsy
GBS: Guillain-Barre syndrome
(MRC: Medical research council
IGRA: Interferon-gamma Release Assay
ADEM): Acute disseminated encephalomyelitis
MRI: Magnetic Resonance Imaging
CT: Computed tomography
IV: Intravenous
